# Marginal Gap of Pre‐Cemented Endocrowns: A Systematic Review of Measurement Methods and the Influence of Fabrication Method and Crown Material

**DOI:** 10.1002/cre2.70152

**Published:** 2025-05-26

**Authors:** James Dudley, Taseef Hasan Farook

**Affiliations:** ^1^ Adelaide Dental School The University of Adelaide Adelaide South Australia Australia

**Keywords:** endorestoration, fit discrepancy, indirect endodontic filling, measurement techniques

## Abstract

**Objective:**

The aim of this systematic review was to analyze the existing literature on the methods used for in vitro marginal gap measurement of pre‐cemented endocrowns and determine whether the fabrication method and material used influenced the marginal gap.

**Materials and Methods:**

Systematic screening was conducted until January 2025 using EBSCO Host, Scopus, PubMed, and Web of Science databases. This systematic review followed PRISMA guidelines, including in vitro studies on marginal gaps in single‐unit pre‐cemented endodontic crowns while excluding in vivo and virtual assessments, and studies analyzing preformed and implant‐supported crown assessments. The quality of the selected studies was evaluated using the Joanna Briggs Critical Appraisal Checklist. Welch's *t*‐test and ANOVA were used to analyze differences in marginal gaps across the included variables.

**Results:**

Twenty‐eight studies were included for analysis. The mean marginal gap for endocrowns across all included studies was 82.75 ± 33.10 µm. Endocrowns fabricated on maxillary teeth demonstrated a mean marginal gap of 77.01 ± 25.46 µm compared with 85.05 ± 36.04 µm for mandibular teeth (*t* = −0.66, *p* = 0.513). Endocrowns fabricated using computer‐aided design/computer‐aided manufacturing (CAD/CAM) (86.65 ± 38.14 µm) had significantly smaller marginal gaps compared with those produced using conventional impressions (109.37 ± 30.05 µm) (*t* = 2.746, *p* = 0.038). Seven measurement methods were reported, with significant differences observed in marginal gap values across the different methods (*F* = 4.61, *p* = 0.013). Impression replica and stereomicroscopy were the most used marginal gap methods, used collectively in 19 (68%) of the included 28 studies. Impression replica (95.49 ± 31.57 µm) and stereomicroscopy (63.69 ± 26.81 µm) methods produced significantly different marginal gap measurements (*p* = 0.003). There were no significant differences in marginal gap between endocrown materials (*p* = 0.122), with marginal gaps ranging from 60.93 ± 26.66 µm in the less explored polymer‐infiltrated ceramic to 95.21 ± 34.07 µm in the more frequently studied zirconia. Lithium disilicate remained the most heavily researched material with a pooled marginal gap of 84.04 ± 32.91 µm across all included studies.

**Conclusion:**

The choice of measurement technique significantly influenced the reported marginal gap values, with impression replica, stereomicroscopy, and 3D superimposition being the most commonly used methods. While the fabrication method had a notable impact on marginal gaps in vitro, the choice of crown material did not.

## Clinical Significance

Marginal gaps were mostly unaffected by tooth form but were smaller with CAD/CAM preparations. The method used for measurement likely impacted results, with stereomicroscopy reporting smaller gaps than impression replica and digital superimposition techniques. Despite lithium disilicate being the most used, crown material choice showed no consistent effect on marginal gaps.

## 1 Introduction

Endocrowns, also known as one‐piece endodontic crowns, are all‐ceramic full‐coverage restorations that differ from conventional single‐unit crowns by incorporating an integrated intracoronal extension that extends into the pulp chamber of a root‐filled posterior tooth (Mannocci et al. 2021). Endocrowns have gained recognition as a feasible restorative option for endodontically treated premolars and molars (Mannocci et al. 2022). The preparation requirements for endocrowns facilitate preservation of residual tooth structure, as post space preparation and placement, and preparation of a ferrule design, are avoided (Mannocci et al. 2022). However, potentially technique‐sensitive adhesive luting is required, including proper isolation of the prepared tooth.

“Marginal gap” is the space between the axial wall of the prepared tooth and the internal surface of the restoration at the margin (Holmes et al. 1989). The marginal fit describes how precisely the crown conforms to the edge of the tooth, thereby affecting the overall size of the marginal gap. Marginal fit influences the success of crowns and is just as important as esthetics and fracture resistance (Contrepois et al. 2013; Alqahtani 2017; Nakamura et al. 2000). The most important factors influencing marginal fit of a restoration include preparation design, finish line location, restorative material used for the crown, method for recording impressions, and crown fabrication method (Shah et al. 2022; Li et al. 2017; Tsirogiannis et al. 2016; Kocaağaoğlu et al. 2017; Yu et al. 2019; Asavapanumas and Leevailoj 2013; Usta Kutlu and Hayran 2024). An exact marginal fit reduces the risk of plaque accumulation and microleakage‐related hypersensitivity, whereas an unsatisfactory marginal fit may result in periodontal disease, caries, pulpitis, and increased cement exposure to the oral environment (Contrepois et al. 2013; Nakamura et al. 2000; Souza et al. 2012).

Clinical marginal gaps of up to 120 µm (Mclean and von 1971) have been deemed satisfactory for conventional crowns, founded on in vitro measurements using polyether rubber films of restorations seated in vivo. Contemporary investigations have recommended that smaller gaps are acceptable, such as ≤100 µmm (Reich et al. 2008; Davis 1988), ≤90 µm (Memari et al. 2019), and ≤75 µm (Hung et al. 1990). In contrast, in a recent comparison of endocrowns fabricated from three different computer‐aided design and computer‐aided manufacturing (CAD/CAM) materials, Taha et al (Taha and Hatata 2024) proposed that marginal gaps of up to 160 µm can be clinically acceptable. Typically, CAD/CAM is used to fabricate endocrowns from lithium disilicate, zirconia, hybrid ceramics, and resin composites within in vitro analyses (Beji Vijayakumar et al. 2021). Past research assessing marginal gaps relied on varying materials, construction methods, and measurement methods (Nawafleh et al. 2013; Paul et al. 2020). Based on evaluations of traditional all‐ceramic crowns, there is no established consensus on the optimal marginal gap. Instead, the focus remains on determining what is considered clinically acceptable; a threshold that is still highly subjective (Sanches et al. 2023).

The methods used for measuring marginal gaps can be classified as 2‐dimensional (2D) or 3‐dimensional (3D), and destructive (DE), or non‐destructive (ND). The primary methods are direct view microscopy (2D, ND), scanning electron microscopy (3D, ND), impression replica (2D, ND), cross‐sectioning (2D, DE), microcomputed tomography (3D, ND), and 3D superimposition (3D, ND). Each method has its own strengths and weaknesses. Studies comparing the precision of scanning electron microscopy and light microscopy in evaluating crown marginal gap have yielded contradictory results (Schmalz et al. 1995; Groten et al. 1997). Numerous reviews have investigated the factors influencing the assessment of crown marginal gap, but a thorough comparison of the different measurement methods has not been conducted (Tsirogiannis et al. 2016; Hung et al. 1990; Nawafleh et al. 2013; Sanches et al. 2023; Hasanzade et al. 2021a; Kandavalli et al. 2022; Van den Breemer et al. 2015).

### 1.1 Study Rationale

Contemporary systematic reviews and meta‐analyses have shown high clinical success rates for endocrowns in molars (72%–99%) and premolars (68%–100%) over a follow‐up duration spanning 3–19 years, with no notable distinction between tooth types (Mannocci et al. 2022; Thomas et al. 2020). Root‐filled molars restored using endocrowns exhibited similar survival rates compared with complete coverage crowns, with survival rates exceeding 90% after seven (Fages et al. 2017) and 10 years (Otto and Mörmann 2015), respectively. Furthermore, research has shown that endocrowns and conventional post‐retained crown restorations exhibit similar survival and success rates (Al‐Dabbagh 2021). Yet previous reviews on endocrowns have predominantly focused on individual tooth survival and success (Thomas et al. 2020; Al‐Dabbagh 2021; Sedrez‐Porto et al. 2016; Hiraba et al. 2024), mechanical properties (Beji Vijayakumar et al. 2021; Sedrez‐Porto et al. 2016; Hiraba et al. 2024), and material characteristics (Beji Vijayakumar et al. 2021; Alwadai et al. 2023). While some comparisons have been made with conventional restorations (Govare and Contrepois 2020; Alnajeeli and Gambarini 2019), the marginal gap has largely been treated as a secondary consideration within subgroup analyses (Govare and Contrepois 2020; Alnajeeli and Gambarini 2019). To date, no systematic review has specifically examined the influence of crown material, fabrication method, and measurement method on the marginal gap of endocrowns.

This study aimed to analyze the existing literature on the in vitro methods used to measure the marginal gap of pre‐cemented endocrowns. The following research questions were posed:

1. Does the type of tooth or the fabrication method of an endocrown affect the resultant marginal gap?
2. Does the method of marginal gap assessment influence the reported gap in endodontic crowns?
3. Does the choice of crown material impact the final pre‐cementation marginal gap of endocrowns?

## 2 Methods

This systematic review was performed in accordance with the Preferred Reporting Items for Systematic Reviews (PRISMA) guidelines. The study protocol was registered using Open Science Framework (https://osf.io/abjvw).

### 2.1 Eligibility Criteria

This systematic review followed the Preferred Reporting Items for Systematic Reviews and Meta‐Analyses (PRISMA) guidelines. The inclusion criteria targeted in vitro studies that evaluated marginal gaps in complete coverage single endodontic crowns, before cementation. Eligible studies needed to provide a comprehensive methodology for gap assessment, including the number of measurements made. Crown types considered included laboratory‐fabricated crowns made of any material, provided they featured an access cavity extension representative of a single‐unit endodontic restoration.

The exclusion criteria eliminated in vivo studies on complete coverage all‐ceramic crowns, metal‐ceramic crowns, all‐metal crowns, or crowns constructed by layering secondary materials onto cast alloys and placed on independently built‐up and prepared abutments. Studies evaluating crowns virtually without a physical access cavity component were also excluded. Studies assessing the trueness and precision of different scanners or scanning techniques without making a physical marginal gap measurement were excluded. Additionally, studies examining marginal gaps in preformed metal crowns, pediatric crowns, fixed partial dentures, or implant‐supported prostheses were excluded. Studies relying solely on manual visual or tactile assessments of marginal gaps or those focusing exclusively on internal fit without addressing marginal gaps were also deemed ineligible.

### 2.2 Search Strategy

Data were gathered from the following databases: Scopus, PubMed. gov, EBSCOHost Dentistry & Oral Sciences Source (DOSS), and Web of Science (WoS). Web of Science encompasses various indexed databases, including the Web of Science Core Collection, Current Contents Connect, Derwent Innovations Index, KCI‐Korean Journal Database, MEDLINE, Russian Science Citation Index, and SciELO Citation Index, with English translations provided where relevant. Searches were conducted in February 2025 without any restrictions on publication dates but limited only to peer‐reviewed literature in EBSCOHost. In Scopus and Web of Knowledge, field‐specific filters were applied to narrow the focus to material sciences in dentistry and healthcare.

The search process was carried out independently by two reviewers (JD and THF). The approach combined keyword‐based logic grids, Boolean operators, and manual reviews of reference lists from related studies. An example search query used for EBSCOHost DOSS is provided below.

(TI(Fit OR Gap* OR Space OR Distance OR Length* OR Accurac* OR Precision) OR AB(Fit OR Gap* OR Space OR Distance OR Length* OR Accurac* OR Precision)) AND (TI(“internal margin*“ OR “internal discrepanc*“ OR “margin* adaptation*“ OR “cervical margin*“ OR preparation OR “margin* integrity” OR “margin* opening*“ OR “edge gap*“ OR “margin* gap*“) OR AB(“internal margin*“ OR “internal discrepanc*“ OR “margin* adaptation*“ OR “cervical margin*“ OR preparation OR “margin* integrity” OR “margin* opening*“ OR “edge gap*“ OR “margin* gap*“)) AND (TI(Endocrown OR “Endodontic Crown” OR “Endorestoration” OR “overlay crown” OR “Endo‐restoration”) OR AB(Endocrown OR “Endodontic Crown” OR “Endorestoration” OR “overlay crown” OR “Endo‐restoration”)).

### 2.3 Selection of Studies and Extraction Process

A systematic review platform (Covidence. org; Veritas Health Innovations Ltd., Melbourne, Australia) was used to screen eligible manuscripts, allowing for automated duplicate removal and ensuring consensus among reviewers. Any disagreements were resolved before advancing eligible articles for further review, ensuring complete agreement on the final selection. The quality of the included studies was evaluated by a prosthodontist with over 15 years of clinical experience (JD) using the Joanna Briggs Institute Critical Appraisal Checklist for Analytical Cross‐Sectional Studies. Notably, no studies were excluded solely based on the quality assessment results.

The following parameters were recorded for analysis: the number of endocrowns assessed, underlying tooth structure, tooth type measured, material used for canal obturation, endocrown material, method of endocrown fabrication, technique used for measuring marginal gaps, number of marginal gap measurements per endocrown, mean range of marginal gap measurements (in µm) in any direction, and mean marginal gap (in µm, rounded to the nearest whole number).

### 2.4 Quality Assessment

The quality of the included studies was evaluated by a prosthodontist with over 15 years of clinical experience (JD) using the Joanna Briggs Institute Critical Appraisal Checklist for Analytical Cross‐Sectional Studies. Notably, no studies were excluded solely based on the quality assessment results.

### 2.5 Statistical Analysis

Statistical analyses were conducted using SPSS software (version 26; IBM Corp). The Shapiro‐Wilk test was applied to assess normality. Welch's *t*‐test was used to evaluate the impact of maxillary versus mandibular teeth on marginal gaps, the differences between plastic and natural human teeth in reported gaps, and the marginal gaps resulting from conventional impressions versus intraoral scans. A Welch one‐way ANOVA was performed to compare the overall means of marginal gaps across different measurement techniques and materials, followed by a Games‐Howell pairwise comparison. Robust Equality of Means was used to test the impact of data distribution on group outcomes.

## 3 Results

**Figure 1:**
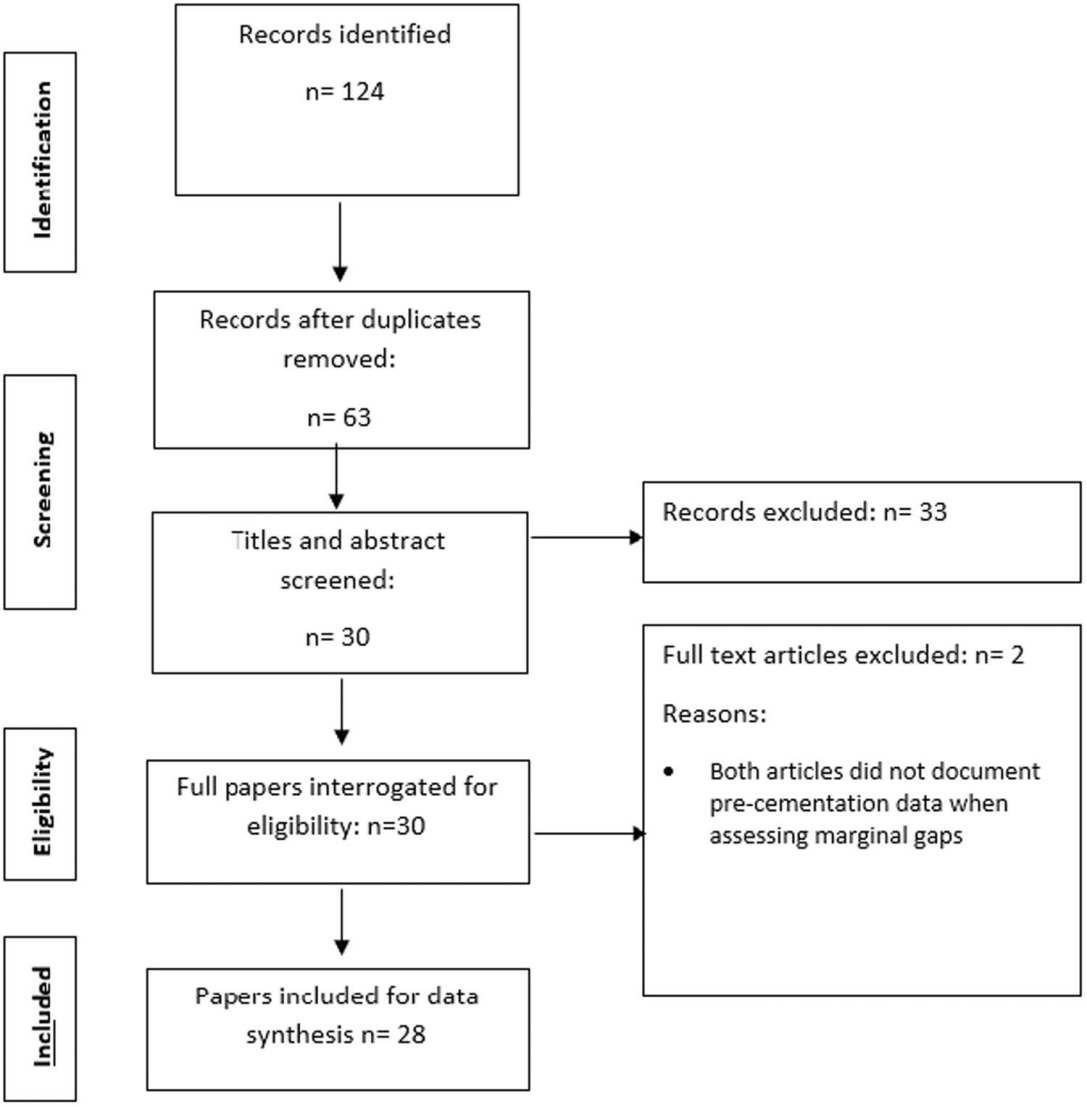
PRISMA flowchart showing search results.

Twenty‐eight studies (Taha and Hatata 2024; Ardekani et al. 2024; Falahchai et al. 2021; Hezavehi et al. 2024; Falahchai et al. 2025; Gupta et al. 2025; Farghal et al. 2024; Jalalian et al. 2024; Mehta et al. 2024; Xiao et al. 2024; Akhlaghian et al. 2024; Elashmawy and Elshahawy 2023; El‐Farag et al. 2023; Hajimahmoodi et al. 2023; Jamshidi et al. 2023; Mertsöz et al. 2023; Suliman and Rayyan 2023; Abduljawad and Rayyan 2022; Bamajboor and Dudley 2022; Zheng et al. 2022; Amini et al. 2021; El Ghoul and Salameh 2021; El Ghoul et al. 2020; Godil et al. 2021; Hasanzade et al. 2021b; Zimmermann et al. 2019; Taha et al. 2018; Gaintantzopoulou and El‐Damanhoury 2016) were included for analysis as illustrated in the PRISMA flowchart (Figure 1). The overall agreement between the two reviewers was ĸ=0.78 before conflict resolution. A total of 1,197 endocrowns were evaluated, with an average of 42.7 ± 25.7 endocrowns per study. Interestingly, 75% of the corresponding authors of the included studies originated from the Middle East (with 35.7% from Iran), and 14.2% from Asia. The mean marginal gap for endocrowns across all included studies was 82.75 ± 33.10 µm. The mean number of marginal gap measurement points made per endocrown was 11.88. The findings from all included studies are presented in Table 1.

**Table 1:** Summary of marginal gap outcomes reported on pre‐cemented endocrowns (*N* = 28).

Author, year	Location	Number of endocrowns assessed	Underlying structure	Tooth form measured	Material used for canal obturation	Endocrown Material	Method of endocrown construction	Technique for measuring marginal gap	Number of marginal gap measurements per endocrown	Mean range of marginal gap measurements (in µm) in any direction		Mean marginal gap (in µm, rounded to nearest whole number)
Falahchai et al. (2025)	Iran	17	Human teeth	Mandibular first molar	Unspecified	Lithium disilicate Zirconia Zirconia reinforced Lithium silicate	CAD/CAM	Replica technique	4	Lithium disilicate	102 ± 11.4 to 105.1 ± 17.1	102
										Zirconia	91 ± 16.4 to 93.1 ± 15.1	
										Zirconia reinforced Lithium silicate	110.0 ± 11.2 to 111.0 ± 19.1	
Gupta et al. (2025)	India	30	Plastic teeth	Mandibular first molars	Not applicable	Lithium disilicate	CAD/CAM	Stereomicroscopy	20	With ferrule	58.9 to 87.5	65
										Without ferrule	46.5 to 66.5	
Ardekani et al. (2024)	Iran	68	Human teeth	Mandibular first molars	Unspecified	Zirconia	Conventional impression with CAM Intraoral scanning with CAM	Replica technique	4	Conventional impression with CAM	101.2 ± 15.9 to 103.9 ± 14.2	92
										Intraoral scanning with CAM	71.2 ± 15.6 to 91.6 ± 14.7	
Farghal et al. (2024)	Yemen	60	Human teeth	Mandibular first molars	Protaper System obturation	Lithium disilicate Zirconia	CAD/CAM	Replica technique	4	Lithium disilicate	90.3 to 101.2	104
										Zirconia	98.3 to 122.8	
Hezavehi et al. (2024)	Iran	40	Human teeth	Maxillary molar	Unspecified	Lithium disilicate Polymer‐infiltrated ceramic	CAD/CAM	Replica technique	4	Lithium disilicate	100.3 ± 6.2	106
										Polymer‐ infiltrated ceramic	112.2 ± 5.0	
Jalalian et al. (2024)	Iran	24	Human teeth	Mandibular first molar	Diadent gutta percha with AH‐Plus sealer	Lithium disilicate Zirconia reinforced lithium silicate	CAD/CAM	Stereomicroscopy	12	Lithium disilicate	43.8 ± 13.3	56
										Zirconia reinforced lithium silicate	67.5 ± 11.2	
Singh 2024	India	42	Human teeth	Mandibular molar	Protaper system obturation	Lithium disilicate Resin nanoceramics Resin polymer	Conventional impression with CAM	Scanning electron microscopy	8	Lithium disilicate	118.2 ± 2.3	97
										Resin nanoceramics	46.3 ± 8.2	
										Resin polymer	125.9 ± 4.5	
Taha and Hatata (2024),	Egypt	30	Human teeth	Mandibular third molars	Unspecified	Lithium disilicate Zirconia Ceramic	CAD/CAM	CBCT	6	Lithium disilicate	92 ± 15.5	162
										Zirconia	200 ± 54.6	
										Ceramic	193 ± 25.8	
Xiao et al. (2024)	China	52	Plastic teeth	Mandibular first molar	Not applicable	Zirconia Resin polymer	CAD and additive manufacturing CAD and milling	3D superimposition	—	*Additive manufacturing*		102
										Zirconia	115.9 ± 24.8	
										Resin polymer	101.5 ± 16.3	
										*Milling*		
										Zirconia	101.6 ± 18.6	
										Resin polymer	87.7 ± 10.2	
Akhlaghian 2024	Iran	40	Human teeth	Maxillary molar	Meta Biomed Gutta Percha and AH26 sealer	Lithium disilicate	Conventional impression with CAM Intraoral scanning with CAM	Stereomicroscopy	1	Conventional impression with CAM	130.3 ± 7.9	109
										Intraoral scanning with CAM	87.09 ± 4.9	
Elashmawy and Elshahawy (2023)	Egypt	72	Human teeth	Mandibular first molars	Protaper System obturation	Lithium disilicate Zirconia Polymer‐ infiltrated ceramic PEEK	CAD/CAM	Cross section of six randomly selected specimens	4	Lithium disilicate	45.2 ± 9.5	57
										Zirconia	63.9 ± 10.8	
										Polymer‐ infiltrated ceramic	37.6 ± 7.1	
										PEEK	83.0 ± 9.5	
El‐Farag et al. (2023)	Egypt	40	Human teeth	Maxillary first molars	Race 25 mm NiTi Rotary system	Zirconia Resin Nanoceramic Polymer‐ infiltrated ceramic PEKK	CAD/CAM	Replica technique followed by microscopy	36	Zirconia	100.3 ± 3.4	83
										Resin Nanoceramic	69.3 ± 3.6	
										Polymer‐ infiltrated ceramic	83.2 ± 5.8	
										PEKK	80.6 ± 3.6	
Hajimahmoodi et al. (2023)	Iran	40	Plastic teeth	Mandibular first molar	Not applicable	Lithium disilicate Zirconia reinforced lithium silicate	CAD/CAM	Replica technique	4	Lithium disilicate	69.0 ± 8.9 to 77.5 ± 10.7	80
										Zirconia reinforced lithium silicate	84.8 ± 15.0 to 89.2 ± 31.0	
Jamshidi et al. (2023)	Iran	20	Plastic teeth	Mandibular first molar	Unspecified	Lithium disilicate Resin polymer	Conventional Heat press production for lithium disilicate CAD/CAM for resin polymer	Stereomicroscopy	8	Lithium disilicate	99.7 ± 4.6	109
										Resin polymer	117.7 ± 2.9	
Mertsöz et al. (2023)	Turkey	120	Plastic teeth	Maxillary first premolar	Not applicable	Polymer‐ infiltrated ceramic Resin nanoceramics	CAD/CAM	Replica technique	4	Polymer‐ infiltrated ceramic	36.5 ± 12.0	32
										Resin nanoceramics	27.6 ± 4.3 to 33.6 ± 8.6	
Suliman and Rayyan (2023)	Saudi Arabia	40	Human teeth	Mandibular molar	Protaper Gold system obturation	Lithium disilicate	CAD/CAM	Stereomicroscopy	20	13.1 ± 7.1 to 46.3 ± 21.2		24
Abduljawad and Rayyan (2022)	Saudi Arabia	40	Human teeth	Mandibular first molar	Protaper system obturation	Lithium disilicate	Conventional impression with heat press production Conventional impression with CAM Intraoral scanning with CAM	Micro‐CT	28 (from 14 slices)	Conventional impression with heat press production	150 ± 35	127
										Conventional impression with CAM	110 ± 24	
										Intraoral scanning with CAM	120 ± 27	
Bamajboor and Dudley (2022)	Australia	20	Human teeth	Mandibular molar	unspecified	Zirconia	CAD/CAM	Stereomicroscopy	12	38.6 to 61.8		47
Zheng et al. (2022)	China	120	Plastic teeth	Mandibular first molar	Not applicable	Zirconia reinforced lithium silicate Resin nanoceramic	CAD/CAM	Micro‐CT	46	*At 30 µm cement space*		69
										Zirconia reinforced lithium silicate	144.7 ± 22.4 to 150.1 ± 19.0	
										Resin nanoceramic	170.4 ± 26.0 to 174.4 ± 22.8	
										*At 60 µm cement space*		
										Zirconia reinforced lithium silicate	52.4 ± 6.6 to 59.9 ± 7.9	
										Resin nanoceramic	79.2 ± 11.5 to 82.7 ± 18.0	
										*At 120 µm cement space*		
										Zirconia reinforced lithium silicate	81.7 ± 14.6 to 82.2 ± 10.9	
										Resin nanoceramic	103.6 ± 7.8 to 105.4 ± 13.6	
Amini et al. (2021)	Iran	28	Human teeth	Maxillary first molar	Unspecified	Zirconia Zirconia reinforced lithium silicate	CAD/CAM	Replica technique and stereomicroscopy	12	Zirconia	58.3 ± 8.4	53
										Zirconia reinforced lithium silicate	54.1 ± 9.0	
El Ghoul et al. (2020)	Lebanon	30	Human teeth	Mandibular first molar	Protaper system obturation	Lithium disilicate	Conventional heat press CAD/CAM	Replica technique	8	Conventional heat press	143.6 ± 9.0	118
										CAD/CAM	91.6 ± 5.0	
Falahchai 2021	Iran	22	Human teeth	Mandibular first molar	Protaper system obturation	Zirconia	Conventional impression with CAM Intraoral scanning with CAM	Stereomicroscopy	8	Conventional impression with CAM	74.0 ± 8.0	72
										Intraoral scanning with CAM	70.0 ± 11.0	
Godil et al. (2021)	Italy	20	Plastic teeth	Mandibular first molar	Not applicable	Lithium disilicate PEEK	CAD/CAM	Stereomicroscopy	4	Lithium disilicate	56.5 ± 6.1	69
										PEEK	81.3 ± 10.9	
Hasanzade et al. (2021a)	Iran	36	Plastic teeth	Maxillary first molar	Not applicable	Lithium disilicate Zirconia reinforced lithium silicate Polymer‐ infiltrated ceramic	CAD/CAM	3D superimposition	—	Lithium disilicate	69.2 ± 23.5	73
										Zirconia reinforced lithium silicate	77.5 ± 13.4	
										Polymer‐ infiltrated ceramic	71.0 ± 31.8	
El Ghoul et al. (2020)	Lebanon	40	Human teeth	Mandibular molars	Flowable composite obturation	Lithium disilicate Zirconia Resin nanoceramic Resin polymer	CAD/CAM	Replica technique	4	Lithium disilicate	104.8 ± 14.1	140
										Zirconia	114.7 ± 21.5	
										Resin nanoceramic	143.0 ± 21.7	
										Resin polymer	196.7 ± 33.7	
Zimmermann et al. (2019)	Switzer‐land	30	Plastic teeth	Maxillary first molar	Not applicable	Zirconia reinforced lithium silicate Ceramic Resin Nanoceramic	CAD/CAM	3D superimposition	—	Zirconia reinforced lithium silicate	99.6 ± 23.7	80
										Ceramic	88.9 ± 7.7	
										Resin Nanoceramic	131.0 ± 26.5	
Taha et al. (2018)	Egypt	40	Human teeth	Mandibular first molar	Protaper system obturation	Lithium disilicate Polymer‐ infiltrated ceramic Zirconia reinforced lithium silicate Resin Nanoceramic	CAD/CAM	Stereomicroscopy	20	Lithium disilicate	36.9 ± 24.4	42
										Polymer‐ infiltrated ceramic	47.0 ± 26.6	
										Zirconia reinforced lithium disilicate	45.8 ± 16.6	
										Resin Nanoceramic	39.4 ± 17.8	
Gaintantzopoulou and El‐Damanhoury (2016)	United Arab Emirates	36	Plastic teeth	Mandibular first molar	Not applicable	Polymer‐ infiltrated ceramic	CAD/CAM	Micro‐CT	16 (from 8 slices)	40.6 ± 4.1 to 59.4 ± 9.6		47

Abbreviations: CAD/CAM, Computer Aided Design/Computer Aided Manufacturing; CBCT, Cone Beam Computed Tomography.

A critical appraisal using the JBI Checklist revealed that most studies failed to identify and manage confounding factors inherent to the measurement methods employed. This was particularly evident in responses to the appraisal items: “Were confounding factors identified?” and “Were strategies to deal with confounding factors stated?” A key concern related to the measurement of marginal gaps, specifically, whether a similarly trained operator would obtain comparable results. Few, if any, studies included multiple operators or outlined a standardization protocol. This lack of standardization may also extend to the question, “Were the outcomes measured in a valid and reliable way?” While such methodological limitations could have influenced the measurement of marginal gaps, they were considered inherent to in vitro endocrown studies and were observed, to some degree, across all included studies.

### 3.1 The Effect of Tooth Form and Fabrication Method on Marginal Gap

Endocrowns were fabricated on either plastic teeth or extracted human teeth. When equal variances were not assumed, the mean marginal gap for plastic teeth was 72.6 ± 22.81 µm compared with 88.39 ± 37.02 µm for extracted human teeth. Welch's *t*‐test indicated no statistically significant difference between the two groups (*t* = −1.39, *p* = 0.175). The mean marginal gap for endocrowns constructed on maxillary teeth was 77.01 ± 25.46 µm compared with 85.05 ± 36.04 µm for mandibular teeth. Welch's *t*‐test revealed no statistically significant difference (*t* = −0.66, *p* = 0.513) when equal variances were not assumed.

The marginal gap measurements were compared between endocrowns fabricated using conventional impression techniques and those produced via 3D scanning in CAD/CAM workflows. Some studies included multiple test groups that utilized both methods. Endocrowns fabricated using CAD/CAM exhibited a mean marginal gap of 86.65 ± 38.14 µm which compared with those produced using conventional impressions (109.37 ± 30.05 µm). A Welch's t‐test for unequal variances demonstrated a statistically significant difference between the groups (*t* = 2.746, *p* = 0.038).

### 3.2 Method of Marginal Gap Assessment and Reported Marginal Gaps

**Figure 2:**
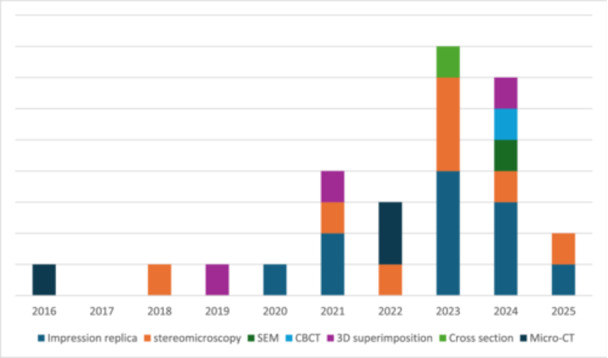
Distribution of measurement methods implemented across the years.

Seven methods were used for measuring marginal gaps in endocrowns: impression replica, stereomicroscopy, scanning electron microscopy, cone beam computed tomography, 3D superimposition, cross‐sectioning, and microCT. Impression replica and stereomicroscopy were the most used marginal gap methods, used collectively in 20 of the included 28 studies. The methods used by year of publication are presented in Figure 2. A Robust Test of Equality of Means indicated significant differences across the methods (F = 4.73, *p* = 0.012) indicating a robust unequally distributed data set. The results are detailed in Table 2 while Table 3 provides a pairwise comparison of the different measurement techniques. Impression replica (95.49 ± 31.57 µm) and stereomicroscopy (63.69 ± 26.81 µm) methods produced significantly different marginal gap measurements (*p* = 0.003). The significance of these findings should be interpreted with some caution, given the imbalance in the number of endocrowns assessed and the studies employing each method.

**Table 2:** Analysis of variance across the marginal gap measurement methods.

Method	N. as	Mean ± SD (in µm)	Confidence Interval		F stat	P*
			Lower bound	Upper bound		
Impression replica	33	95.49 ± 31.57	84.29	106.68	6.40	< 0.001*
Stereomicroscopy	24	63.69 ± 26.81	52.37	75.01		
SEM	3	96.80 ± 43.90	12.26	205.83		
CBCT	3	161.66 ± 60.43	11.54	311.80		
3D superimposition	10	94.39 ± 19.64	80.33	108.44		
Cross section	4	57.43 ± 20.32	25.09	89.76		
Micro‐CT	17	103.92 ± 41.99	82.33	125.51		

*Note:* Welch's 1‐way ANOVA. N.as N. as (Number of assessments); pooled number of measurements per method, aggregated across variations in materials or fabrication methods evaluated within included studies.

Abbreviation: SD, Standard Deviation.

* Significant <0.05.

**Table 3:** Pairwise comparison significance (*P* value) outcomes across techniques using Games–Howell assessment.

	Impression replica	Stereo‐microscopy	SEM	CBCT	3D Super‐imposition	Cross section	Micro‐CT
Impression replica	—						
Stereomicroscopy	0.003*	—					
SEM	> 0.999	0.828	—				
CBCT	0.611	0.371	0.738	—			
3D superimposition	> 0.999	0.017*	> 0.999	0.602	—		
Cross section	0.148	0.996	0.766	0.334	0.168	—	
Micro‐CT	0.989	0.027*	> 0.999	0.710	0.983	0.089	—

Abbreviations: CBCT, Cone Beam Computed Tomography; SEM, Scanning Electron microscopy.

### 3.3 Choice of Crown Material and Resultant Marginal Gap

**Figure 3:**
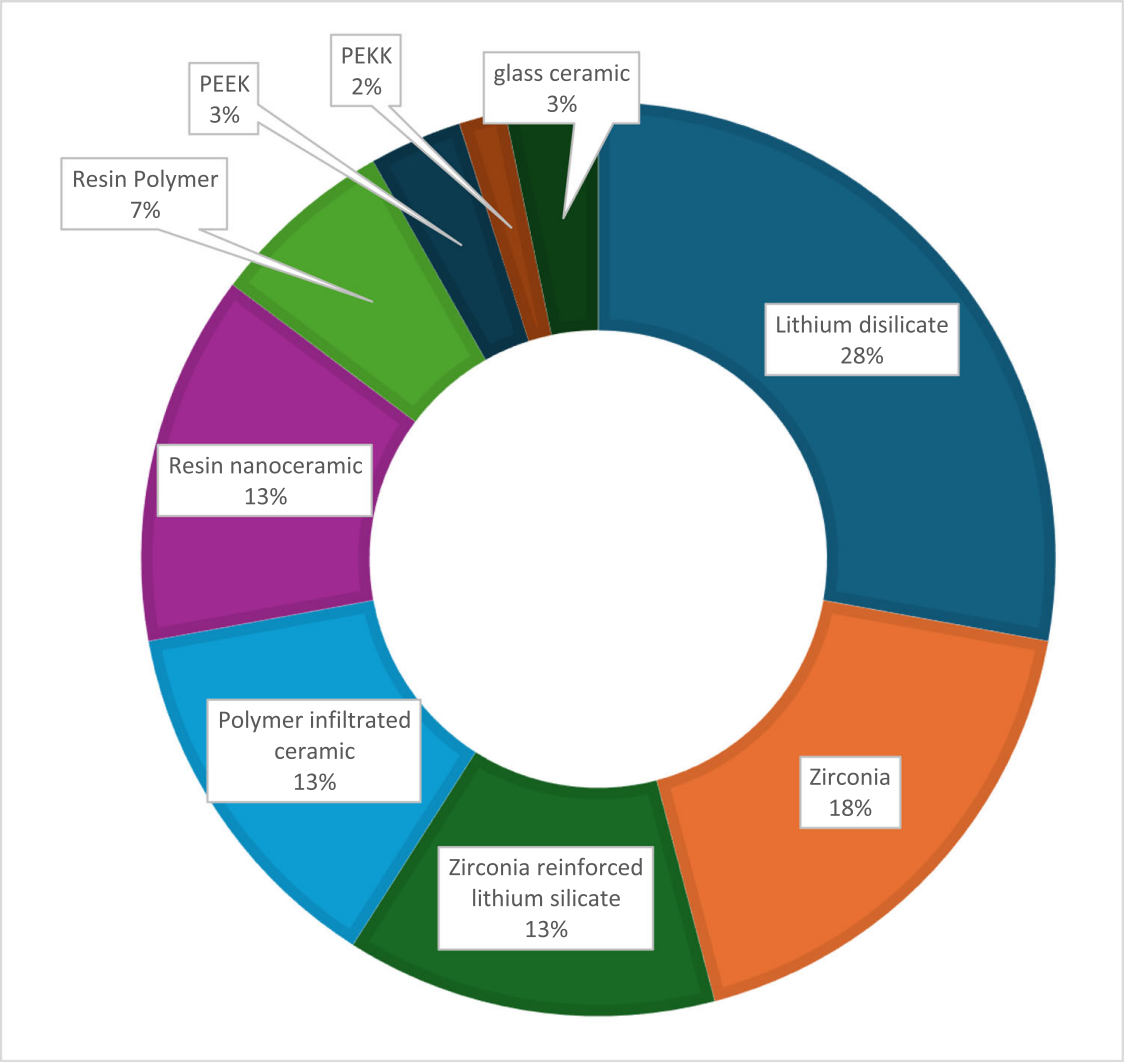
Materials used to fabricate endocrowns in the included studies.

**Figure 4:**
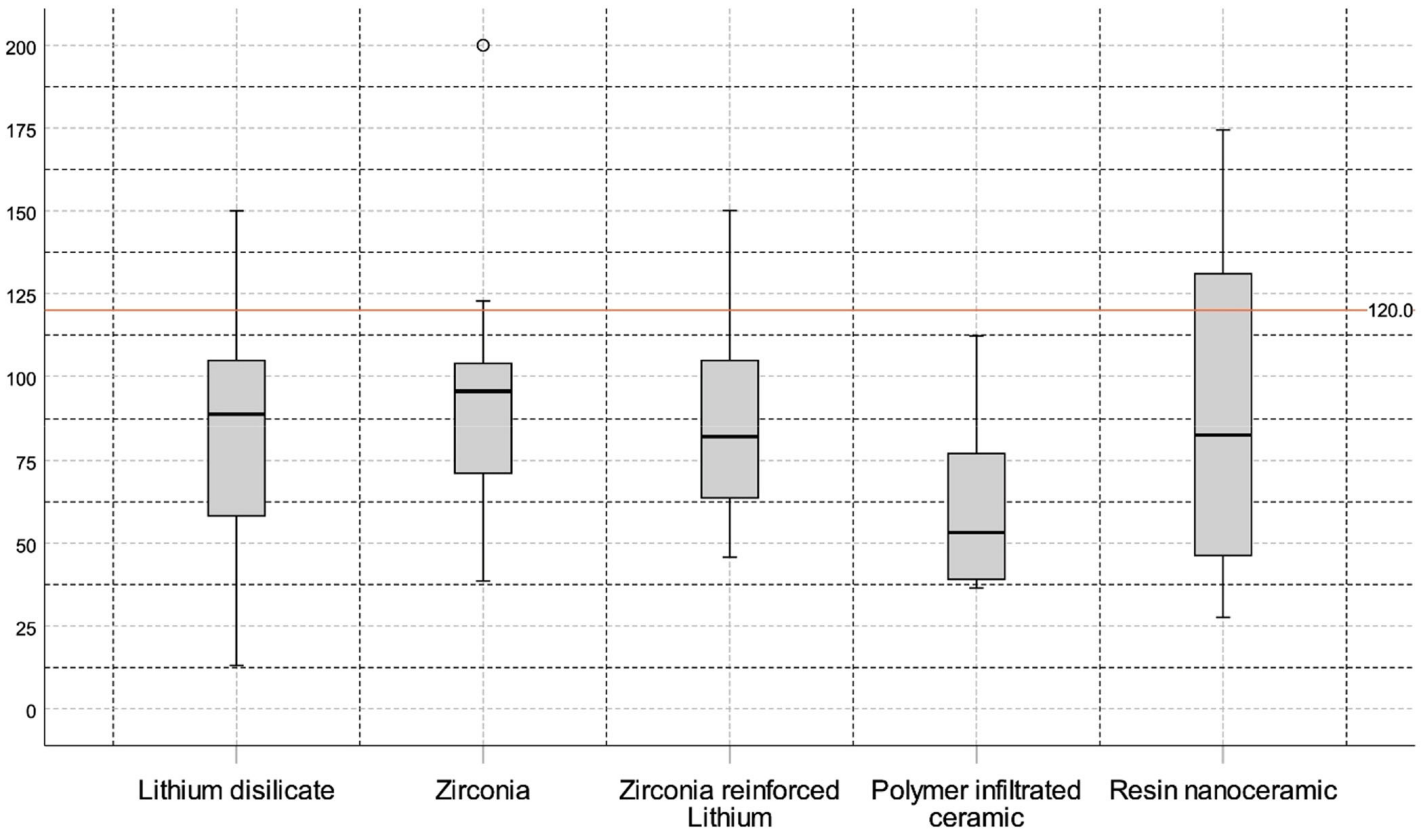
Distribution of reported marginal gaps, in µm (Y‐axis) across the five most documented materials used to fabricate endocrowns (X‐axis).

Figure 3 shows the distribution of materials used to fabricate endocrowns in the included studies. Often, a single study employed an assessment of several different materials. The five most used materials for fabricating endocrowns were lithium disilicate, zirconia, zirconia‐reinforced lithium silicate, polymer‐infiltrated ceramic, and resin nanoceramic. However, the Equality of Means test did not yield a significant result (*p* = 0.122). Material‐specific outcomes are summarized in Table 4. To maintain analytical robustness and account for potential confounding variables, marginal gap values for each material across all 28 studies were pooled, incorporating variations such as conventional milling, different margin designs, variations in taper, varying cement spaces, and pulp chamber forms. Lithium disilicate was the most researched material, with a pooled mean marginal gap of 84.04 ± 32.91 µm across all included studies compared with the next most researched material, zirconia, with a mean marginal gap of 95.21 ± 34.07 µm. However, these findings should be interpreted with caution, as certain materials, such as polymer‐infiltrated ceramics, were examined under less diverse conditions compared with more extensively studied materials such as lithium disilicate and zirconia. To support interpretation, the distribution of reported marginal gaps across the five materials is presented in Figure 4.

**Table 4:** Comparison of the most frequently reported materials in the fabrication of endocrowns.

Materials	N. as	Mean ± SD (in µm)	95% confidence Intervals		F stat	P*
			Lower bound	Upper bound		
Lithium disilicate	30	84.04 ± 32.91	71.76	96.34	1.432	0.231
Zirconia	18	95.21 ± 34.07	78.27	112.16		
Zirconia reinforced lithium silicate	15	87.37 ± 31.41	69.97	104.76		
Resin nanoceramic	13	92.76 ± 50.45	62.27	123.25		
Polymer‐infiltrated ceramic	8	60.93 ± 26.66	38.65	83.23		

*Note:* Welch's 1‐way ANOVA. N. as, pooled number of assessments per material across studies.

Abbreviation: SD, Standard Deviation.

* Significant <0.05.

## 4 Discussion

Endocrowns represent a relatively recent restorative option, combining simplicity of preparation (compared with the alternatives) with the ease of manufacturing a one‐piece esthetic restoration. The first study included in this systematic review assessing marginal gaps of pre‐cemented endocrowns was in 2016, with most included studies conducted in the past 4 years. In the majority of endocrown studies, the marginal gap was reported as one of multiple properties measured that typically encompassed internal adaptation and fracture resistance (Ciobanu et al. 2023). This indicates a move away from traditional philosophy that marginal fit is important for the success of endocrowns ahead of fracture resistance and esthetics (Contrepois et al. 2013; Alqahtani 2017; Nakamura et al. 2000). Alternatively, the measurement of marginal gap may be more challenging to perform requiring specialized and potentially expensive equipment, hence discouraging its investigation. Apart from the generally accepted suggestion of 2‐4 mm of pulp chamber depth preparation (Hasanzade et al. 2021a; Hezavehi et al. 2024; Bamajboor and Dudley 2022), the preparation requirements for endocrowns have not been defined. Correspondingly, the most important factors for endocrown success have not been defined.

### 4.1 Measurement Method and Marginal Gap

While previous research indicated that scanning electron microscopy is more precise than light microscopy (Schmalz et al. 1995), other investigators argued that no significant difference exists in accuracy between the two methods (Groten et al. 1997). Scanning electron microscopy (SEM) may offer more realistic observations of marginal gaps; however, such studies are now considered outdated (Schmalz et al. 1995; Groten et al. 1997). This was reflected in the current review, where only one study employed SEM to analyze crown marginal gaps.

Impression replica and stereomicroscopy were the most used marginal gap methods in the current systematic review. Stereomicroscopy measures at the margin only, is non‐destructive, and measures in two‐dimensions. It is less time‐consuming than other methods and has a reduced chance of error accumulation that may result from the removal and reseating of the crown onto the die (Nawafleh et al. 2013). The use of the impression replica method was first described as a technique in 1983 (Rissin and Wetreich 1983), and subsequently used in early marginal gap analysis research (Davis et al. 1989), then compared with alternative measurement methods (Rastogi and Kamble 2011). The impression replica method records the complete intaglio of the crown, not only at the margin, and is therefore not entirely specific for the purpose of measuring the marginal gap. The impression replica method is popular, non‐destructive, and can be used in vitro and in vivo; however, it is delicate in the handling of thin sections of material (Trifkovic et al. 2012), often difficult to get an intact impression at the margin (Haddadi et al. 2019), and can only be sectioned a limited number of times (Haddadi et al. 2019). The multiple stages involved in the removal and reseating of the crown onto the die have the potential to introduce inaccuracies (Nawafleh et al. 2013).

The impression replica method has been validated for assessing the internal and marginal fit of crowns (Haddadi et al. 2019; Laurent et al. 2008); however, a direct comparison with stereomicroscopy remains lacking, especially since the impression replica technique often involves viewing sectioned material under a microscope. It is unclear whether the smaller marginal gaps reported using stereomicroscopy reflect greater accuracy, as both techniques exhibited larger standard deviations across pooled datasets. Further research is also needed to evaluate how different microscopy techniques affect measurement accuracy (Ayres et al. 2024). Despite the emergence of advanced alternatives like digital superimposition and micro‐CT scanning, the continued use of impression replicas and stereomicroscopy may be attributed to their simplicity and lower cost. Nonetheless, impression replica is highly operator‐dependent, with variability introduced by sectioning angle, impression pressure, and manual measurement. This results in inter‐ and intra‐operator inconsistencies and susceptibility to dimensional degradation, limiting opportunities for reanalysis.

### 4.2 Number of Measurements

Typically, multiple marginal gap measurements are made at convenient locations and averaged to infer the overall crown marginal gap (Holmes et al. 1989; Tan and Dudley 2022; Su and Dudley 2022). A minimum of 50 measurements per crown has been recommended to reduce potential errors, although this recommendation was derived from arithmetic mean calculations related to gap variation and desired precision in a small sample size (Groten et al. 2000). Currently, there is no defined number of measurements required for an accurate assessment of crown marginal gap (Matta et al. 2012; Kwong and Dudley 2020; Dudley and Xu 2024).

The mean number of marginal gap measurement points made per endocrown was 11.88 (maximum was 46), with 15 studies making less than 10 measurements. This finding lies considerably below the historically recommended 50 measurements (Groten et al. 2000). All studies made measurements at locations of convenience, and there is potential that the limited number of measurements made did not accurately represent the actual mean marginal gap of the complete margin circumference. Clinically, this compromises the reliability of marginal gap assessments and may affect decision‐making regarding the fit and long‐term success of endocrowns. Future research should focus on establishing an appropriate number of samples and measurements to ensure meaningful and accurate results instead of relying on the estimations from previous studies or convenience factors.

### 4.3 Endocrown Material and Marginal Gap

Previous research indicates that most comparative studies on endocrown materials are in vitro, with limited clinical evidence supporting material selection. As a result, lithium disilicate ceramics are commonly recommended as the material of choice (El‐Ma'aita et al. 2022). Lithium disilicate was the most extensively studied material, followed by zirconia, and was associated with a slightly smaller mean marginal gap, acknowledging the limitations in reported comparisons. It is favored for its widespread availability, affordability, ease of use, and superior esthetics due to greater translucency compared with zirconia. Although zirconia endocrowns exhibit higher fracture resistance, their tendency to fail in an irreparable manner may limit their clinical desirability (Jalalian et al. 2024).

### 4.4 Geographic Trend

Eighty‐nine percent of the corresponding authors of the included studies were based in the Middle East and Asia. This may be attributed to the lower cost of laboratory‐based manufacturing in these regions compared with Western countries. As such, the popularity and focus of endocrown research may be influenced by geographical and economic factors.

### 4.5 Limitations and Future Recommendations

The current research was an in vitro study limited to pre‐cemented endocrowns. Multiple experimental variables were found in the included studies, such as different construction techniques, preparation dimensions, materials, and measurement methods that limited the statistical analysis, which was evidenced by the relatively large standard deviations. Future research should concentrate on standardizing the method of marginal gap measurement to enable accuracy independent of the method used. Subsequent advancement to measuring marginal gaps in vivo is required to inform clinical decisions.

To improve standardization with in vitro experiments assessing marginal gaps in endocrowns, future research should outline how the underlying tooth specimens were conditioned before tooth preparation. Future experiments may adopt consistent tooth preparation protocols, including uniform pulp floor and canal preparation, axial wall divergence, and finish line geometry. The method of marginal gap measurement should also be standardized, with a preference for reproducible and operator‐independent techniques such as the impression replica method carried out by more than one operator. Alternatively, digital superimposition can be used to minimize manual calibration and reduce human‐induced bias. Consistency in CAD/CAM workflows, with clear reporting of system type, ceramic material, and milling parameters, will further enhance comparability. Lastly, strict adherence to comprehensive reporting guidelines, including sample size justification and statistical methods, can strengthen reproducibility and the overall quality of evidence across studies.

## 5 Conclusions

The following conclusions can be drawn from the current review:

1. Marginal gaps were not influenced by the restored tooth being a maxillary or mandibular posterior tooth. However, preparations using the CAD/CAM approach demonstrated smaller marginal gaps compared with conventional methods.
2. Impression replica and stereomicroscopy were the most frequently used methods for gap measurement. The method of measurement significantly influenced the reported marginal gaps, with impression replica and 3D superimposition yielding comparable values, while stereomicroscopy tended to report smaller gap measurements.
3. Although lithium disilicate was the most commonly used material, the choice of crown material did not have a significant impact on the marginal gap of endocrowns.

These findings should be interpreted with caution due to a lack of standardization across studies and the presence of confounding variables that were not adequately addressed.

## Author Contributions

**James Dudley:** conceptualization, data curation, funding acquisition, investigation, methodology, project administration, resources, supervision, validation, visualization, roles/writing – original draft, writing – review and editing. **Taseef Hasan Farook:** data curation, formal analysis, funding acquisition, investigation, methodology, resources, software, validation, visualization, roles/writing – original draft, writing – review and editing.

## Ethics Statement

The authors have nothing to report.

## Conflicts of Interest

The authors declare no conflicts of interest.

## Data Availability

Data that support the findings of this study are available from the corresponding author upon reasonable request.
